# Influence of Storage on the Antimicrobial and Cytotoxic Activities of a Nisin-biogel with Potential to be Applied to Diabetic Foot Infections Treatment

**DOI:** 10.3390/antibiotics9110781

**Published:** 2020-11-06

**Authors:** Rui Silva Soares, Raquel Santos, Eva Cunha, Luís Tavares, Alexandre Trindade, Manuela Oliveira

**Affiliations:** CIISA-Centro de Investigação Interdisciplinar em Sanidade Animal, Faculdade de Medicina Veterinária, Universidade de Lisboa, 1049-001 Lisboa, Portugal; rmsoares@fmv.ulisboa.pt (R.S.S.); raq.martins.santos@gmail.com (R.S.); evacunha@fmv.ulisboa.pt (E.C.); ltavares@fmv.ulisboa.pt (L.T.); moliveira@fmv.ulisboa.pt (M.O.)

**Keywords:** antimicrobial peptide, nisin, *Staphylococcus aureus*, diabetic foot ulcers, diabetic foot infections, guar gum, cytotoxicity

## Abstract

*Staphylococcus aureus* is the most prevalent pathogen in diabetic foot infections (DFIs). In addition to its ability to express several virulence factors, including the formation of recalcitrant biofilms, *S. aureus* is also becoming increasingly resistant to most antibiotics used in clinical practice. The search for alternative treatment strategies for DFI is urgently needed. Antimicrobial peptides (AMPs), namely, nisin, are emerging as potential new therapeutics for managing DFIs. Our team has developed a nisin-guar gum biogel to be applied to DFIs. In this study, to confirm its future in vivo applicability, we evaluated the influence of four storage temperatures (−20 °C, 4 °C, 22 °C, and 37 °C) during a 24 months storage period on its antimicrobial activity towards DFI *S. aureus*, and its cytotoxicity, to a human keratinocyte cell line. When stored at temperatures below 22 °C, the biogel antimicrobial activity was not significantly influenced by storage duration or temperature. Moreover, nisin incorporated within the guar gum biogel exhibited no significant levels of cytotoxicity on human keratinocyte cells, confirming its potential for DFIs therapeutics. In conclusion, results confirm that the nisin-biogel is a potential candidate to be used as an alternative or complement compound for conventional DFI therapeutics.

## 1. Introduction

Antibiotic resistance is a serious threat to public health, and infections caused by antibiotic-resistant strains are increasingly being reported worldwide [1]. Antimicrobial peptides (AMPs) are emerging as novel therapeutic approaches to overcome the challenges raised by the spreading of antibiotic-resistant bacteria. This diverse group of small peptides can be found in most living organisms as part of their innate immune system and may be used as an alternative to conventional antibiotics [2]. Besides their direct antimicrobial activity against pathogens, AMPs also play a key role in modulating the immune system [3]. Moreover, due to their action mechanisms, bacteria are less likely to develop resistance towards AMPs compared to conventional antibiotics [4,5].

Lantibiotics are a class of AMPs that contain the amino acids lanthionine or methyllanthionine, being produced by Gram-positive bacteria to prevent the multiplication of other microorganisms [6]. Nisin, a type A lantibiotic, is the most well studied and characterized AMP. This small cationic peptide is produced by *Lactococcus lactis* and approved by the Food and Drug Administration, the European Food Safety Authority, the Food and Agriculture Organization, and the World Health Organization as a safe additive. Over the past decades, nisin has made a significant impact in the food industry as a natural biopreservative for use in processed cheeses and heat-treated meat products [7,8,9]. Nisin’s most recently established acceptable daily intake dosage is 1 mg/kg body weight [9].

Nisin’s potent antimicrobial activity against a wide range of pathogens has prompted research towards its application in biomedical fields. Several studies have already demonstrated that the antimicrobial action of nisin also includes clinical isolates [8]. Particularly, a recent study conducted by our team has shown that nisin is able to inhibit and eradicate planktonic and biofilm-organized *Staphylococcus aureus* strains isolated from clinically infected diabetic foot ulcers, including methicillin-resistant and multidrug-resistant strains. Nisin was tested alone and incorporated within a guar gum biogel, to evaluate its efficiency as a delivery system for this AMP [10], and the promising results obtained supported nisin’s application for managing diabetic foot infections (DFIs).

In order to confirm the inhibitory ability and safety of the nisin-biogel formulation as a novel antimicrobial topical therapy, it is mandatory to evaluate the optimal environmental conditions for its storage, especially in terms of time and temperature, and its cellular toxicity potential. The study hereby presented was designed to evaluate nisin’s antimicrobial activity against *S. aureus* DFI isolates after storage at different temperatures during a 24 months period, and to investigate nisin’s cytotoxic activity using a culture of human epidermal keratinocytes.

## 2. Results

### 2.1. Evaluation of Storage Assays

**Figure 1:**
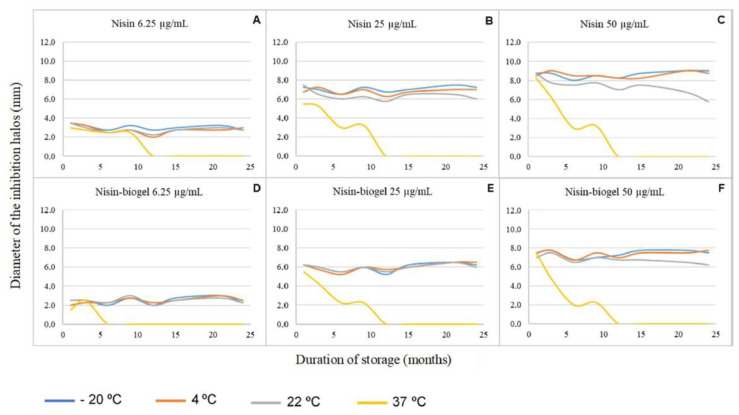
Influence of storage conditions in terms of temperature, duration, and delivery system used on nisin antimicrobial activity against the diabetic foot infection staphylococci under study. Results from all the assays performed. (**A**–**C**) Nisin diluted in water. (**D**–**F**) Nisin diluted in guar-gum biogel.

**Figure 2:**
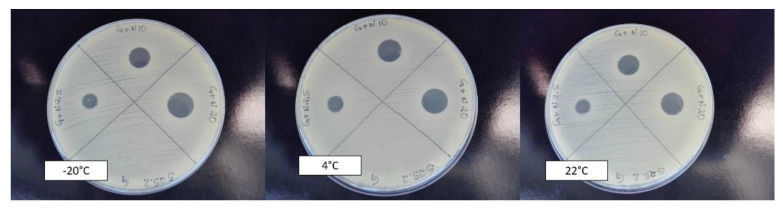
Influence of storage conditions in terms of temperature, time, and delivery system used on nisin antimicrobial activity against the diabetic foot infection staphylococci under study. Antimicrobial activity of the nisin-biogel after 21 months of storage at −20 °C, 4 °C, and 22 °C regarding the isolate Z25.2. G represents the guar-gum control; G + N2.5 represents the biogel supplemented with nisin at 6.25 µg/mL; G + N10 represents the biogel supplemented with nisin at 25.0 µg/mL; and G + N20 represents the biogel supplemented with nisin at 50.0 µg/mL.

Results regarding the influence of different storage conditions on the nisin-biogel antimicrobial activity against DFI staphylococci are summarized in Figure 1 and Figure 2.

For both delivery systems under study (water and guar gum biogel), the inhibition halos diameters were directly proportional to the nisin’s concentration used in each assay. At a concentration of 6.25 µg/mL, no significant differences in nisin’s antimicrobial activity were observed between the AMP suspension in the two delivery systems under study when stored at 4 °C, 22 °C, or 37 °C. In contrast, when stored at −20 °C, the nisin-biogel presented an antimicrobial activity significantly lower (*p* = 0.0029; the difference between means = 0.5938 ± 0.1649 mm) than nisin diluted in sterile water (Figure 1A,D). At a concentration of 25 µg/mL, no significant differences were detected in nisin’s antimicrobial activity between the AMP suspension in the two delivery systems under study when stored at 22 °C and 37 °C. However, when stored at −20 °C and 4 °C, the nisin-biogel exhibited an antimicrobial activity significantly lower (*p* < 0.0001; the difference between means = 1.063 ± 0.1875 mm and *p* = 0.0007; the difference between means = 0.8125 ± 0.1875 mm, respectively) than nisin diluted in sterile water (Figure 1B,E). Similar results were observed for the highest concentration of nisin analyzed, 50 µg/mL, with no significant differences between nisin suspensions in the two delivery systems when stored at 22 °C and 37 °C, and a significantly lower antimicrobial activity presented by the biogel delivery system when stored at −20 °C and 4 °C (*p* < 0.0001; the difference between means = 1.219 ± 0.1826 mm and *p* < 0.0001; the difference between means = 1.188 ± 0.1628 mm, respectively) (Figure 1C,F).

Regarding storage temperatures, no significant differences were observed between nisin’s antimicrobial activity when stored at −20 °C, 4 °C, and 22 °C for the two delivery systems, and all concentrations of nisin were analyzed. However, when stored at 37 °C, nisin’s inhibition halos were significantly smaller (*p* < 0.05), revealing a lower antimicrobial activity when nisin was stored at 37 °C, as compared to storage at the other temperatures analyzed. In fact, at a concentration of 6.25 µg/mL, the mean difference between the inhibition halos produced by nisin stored at 37 °C and by nisin stored at lower temperatures was >1.938 mm for nisin-biogel and >1.469 mm for nisin diluted in sterile water (Figure 1A,D); this difference increased for >4.188 mm for nisin-biogel and for >4.250 mm for nisin diluted in sterile water at a concentration of 25 µg/mL (Figure 1B,E), and for >4.719 mm for nisin-biogel and for >4.750 mm for nisin diluted in sterile water at a concentration of 50 µg/mL (Figure 1C,F).

Regarding the duration of storage, a linear regression analysis showed that for all the nisin’s concentrations and delivery systems under study stored at −20 °C, 4 °C, and 22 °C, the storage period does not influence significantly (*p* > 0.05) nisin’s antimicrobial activity against the DFI staphylococci under study. However, when stored at 37 °C, the storage period significantly influences nisin’s activity (*p* < 0.05). The longer the storage duration, the lower the antimicrobial activity exhibited by nisin. In fact, for all the suspensions under study, nisin did not maintain its activity for more than 12 months when stored at 37 °C.

### 2.2. Evaluation of Nisin Cytotoxicity

**Figure 3:**
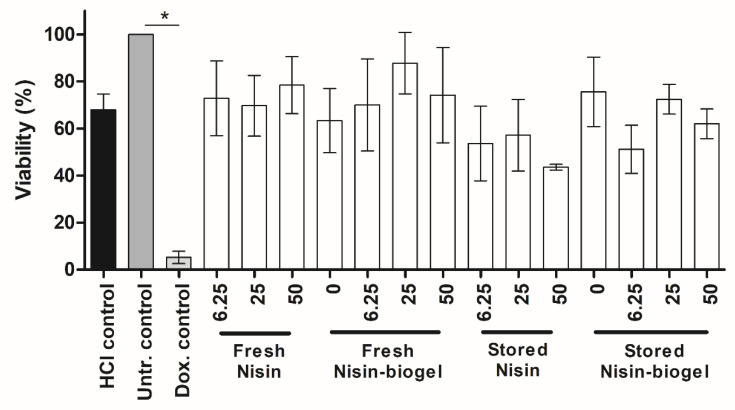
Cytotoxicity of nisin suspensions under study regarding adult human epidermal keratinocyte (HEKa). Comparisons between treatments and HCl control was made using an analysis of variance with the level of significance set at * *p* < 0.05. Concentrations of nisin are expressed in µg/mL. Untr.: Untreated; Dox.: Doxorubicin.

The cytotoxic effects of the nisin suspensions tested on human keratinocyte cells are presented in Figure 3 as a percentage of cell viability as compared to the untreated control. As the nisin stock solution was prepared using 0.02 M HCl, this HCl solution was used in the cytotoxicity assay as solvent control, and all the cytotoxicity results regarding the nisin suspensions under study were compared to this control.

Results show that when treated with the solvent control, the cell viability was slightly lower from the one presented by the untreated control (*p* = 0.0068; the difference between means = 32.06% ± 6.23) and significantly higher from the one presented by the doxorubicin positive control (*p* < 0.0001; the difference between means = 62.72% ± 4.17).

However, regarding the nisin suspensions tested, no significant differences (*p* > 0.05) were observed between their cytotoxicity results and the ones from the solvent control, revealing that nisin did not exhibit cytotoxic effects in the tested concentrations. Also, no significant differences (*p* > 0.05) were observed between the cytotoxicity of the nisin suspensions freshly prepared and of the ones stored at 4 °C for 24 months, showing that long-term storage at 4 °C had no effect on nisin cytotoxicity. Similar results were presented by the analysis of nisin cytotoxicity when incorporated in the delivery systems under study, with no significant differences (*p* > 0.05) being detected between the cell viability when cells were treated with nisin suspensions in sterile water and with nisin suspensions in the guar gum biogel. Therefore, the incorporation of nisin into guar gum gel has no impact on nisin cytotoxicity and may be considered nontoxic.

## 3. Discussion

During the last decades, AMPs have gained an increasing interest as novel potential alternatives for treating a vast array of clinical conditions, particularly those caused by antibiotic-resistant microorganisms. Nisin is a well-known AMP with recognized activity towards gram-positive bacteria, being used as a food preservative for over 50 years and 48 countries concepts [11]. However, despite its demonstrated antimicrobial activity against pathogenic bacteria, including *Bacillus*, *Clostridium*, *Listeria*, and *Streptococcus*, nisin is only used as a food preservative and has currently no therapeutic use [9,12].

Since 2015, our team has been studying the activity of nisin against bacterial isolates collected from infected diabetic foot ulcers, focusing on the potential of topical administration of this peptide. For this reason, nisin’s antimicrobial potential has been evaluated by incorporating this AMP within a guar gum gel, a natural polysaccharide which upon dilution in water forms a jellified formulation suitable for skin application. In spite of both nisin and guar gum being considered safe for human administration [9], the cytotoxic potential of their combined use was still unknown. The study hereby presented determined the most suitable conditions for the storage of the nisin-biogel and evaluated its potentially toxic effects regarding human keratinocyte cells.

Nisin was incorporated in the guar gum gel and stored at four different temperatures for 24 months. Results obtained demonstrated that the biogel delivery system allows nisin to maintain its antimicrobial activity against DFI staphylococci when stored at a wide range of temperatures, namely, between −20 °C and 22 °C. Having in mind that a storage temperature of −20 °C implies a thawing step before applying the nisin-biogel, our recommendation for diabetic patients’ daily utilization is that the supplemented biogel should be stored at 4 °C, the temperature of a conventional domestic fridge.

An adequate antimicrobial compound for topical administration must present low cytotoxic effects on human skin cells. In this study, HEKa cells were exposed to nisin and to nisin-biogel, and their cytotoxicity was evaluated using the MTT cell viability assay, which provides a simple and accurate method to quantify cell viability. The assay is based on converting water-soluble MTT compound to an insoluble formazan product, being observed that only viable cells with an active metabolism, specifically mitochondrial respiration, can convert MTT into formazan. Therefore, the measured optical density is proportional to the number of active metabolic cells, and therefore, viable cells [13].

Studies available on the cytotoxicity of nisin regarding keratinocyte cells are scarce, being observed that results depend on cell type. Kamarajan and colleagues [14] showed that nisin ZP, a naturally occurring variant of nisin, does not induce apoptosis in human oral keratinocytes. Shin et al. [15] reported that human cells present in the oral cavity, mainly gingival fibroblasts, are unaffected by exposure to nisin at anti-biofilm concentrations, showing no signs of apoptotic changes. Moreover, subacute toxicity studies in rats demonstrated that repetitive intravaginal application of nisin induced no morphological changes in vaginal epithelial cells. Additionally, this study by Aranha et al. [16] described no histopathological abnormalities in vaginal tissue or any changes in blood and serum biochemical profiles. However, a previous study by Murinda and colleagues [17] indicated that some bacteriocins, including nisin, can present toxicity regarding colonic and kidney epithelial cells in a dose-dependent manner, and Kamarajan et al. [14] also reported an induced apoptosis dose-dependent in human umbilical vein endothelial cells after exposure to nisin ZP.

Our work evaluated the viability of HEKa cells after exposure to three different concentrations of nisin after incorporation in two different delivery systems, as well as the influence of storage at 4 °C for 24 months on nisin suspensions cytotoxic potential, as this is the temperature that we recommend for the biogel storage. Results from all the suspensions under study were compared to a 0.02 M solution of HCl. While the HCl control presented slight (but significant) cytotoxicity, regarding HEKa cells, no significant differences were observed between the cytotoxicity results from the HCl control and the nisin suspensions tested. Therefore, we can conclude that the cytotoxicity presented by these suspensions is due to the HCl solvent and not to the nisin peptide itself. Further research is necessary to develop strategies to prevent and minimize the toxicity presented by HCl regarding human keratinocyte cells.

Cytotoxicity assay results also demonstrated that the guar gum biogel is a safe delivery system for this peptide, since no significant differences were observed between nisin suspensions diluted in sterile water and those incorporated within the biogel. Additionally, regarding storage duration, results demonstrated that nisin suspensions stored at 4 °C for 24 months presented cytotoxicity levels similar to freshly prepared nisin.

Overall, the data presented in this study shows that, at concentrations up to 50 µg/mL, nisin can be safely administered to human keratinocyte cells. Moreover, the guar gum biogel has proven to be a safe and effective delivery system for this peptide, supporting its use for topical application in treating chronically infected DFUs.

## 4. Materials and Methods

### 4.1. Bacterial Isolates

This study included four *S. aureus* isolates (two *mecA* positive and two *mecA* negative isolates) belonging to a larger bacterial collection of Diabetic Foot Infections isolates obtained from 49 Diabetic Foot Ulcers samples collected in a transversal observational study conducted at four clinical centers in Lisbon, from January 2010 to July 2010 [18]. Isolates were obtained from clinical swab samples collected by the Levine method from infected foot ulcers of hospitalized and ambulatory patients with diabetes mellitus, using conventional microbiological procedures as previously described, after approval by the Faculty of Medicine of the University of Lisbon Research Ethics Committee and the Portuguese Data Protection Authority, and after written informed consent by every patient [18]. Isolates virulence and antibiotic resistance profile were previously characterized [19], as well as their biofilm-forming ability [20] and nisin’s susceptibility profile [10]. Isolates characteristics are summarized in Table 1.

### 4.2. Antimicrobial Peptide Preparation

A nisin stock solution (1000 µg/mL) was prepared as described by Santos et al. (10), by dissolving 1 g of nisin powder (2.5% purity, Sigma-Aldrich, St. Louis, MO, USA) in 25 mL of HCl (0.02 M) (Merck, Darmstadt, Germany), filtered using a 0.22 µm cellulose acetate membrane filter (VWR, Leuven, Belgium) and stored at −20 °C.

A guar gum biogel of 1.5% (*w*/*v*) was also prepared as described by Santos et al. (10) by dissolving 0.75 g of guar gum (Sigma-Aldrich, USA) in 50 mL of deionized sterile water, followed by sterilization by autoclave.

Then, the nisin stock solution was used to prepare a set of nisin dilutions in water or in the guar gum biogel (1:1), aiming to obtain solutions with nisin concentrations of 6.25, 25.0 and 50.0 µg/mL and 0.75% (*w*/*v*) guar gum, which were stored at four different temperatures, representing the average temperatures of freezing (−20 °C), cooling (4 °C) and ambient storage (22 °C) and the average body temperature (37 °C), during a period of 24 months. The guar gum concentration of the biogel allows the formation of a pellicle in the skin, which is essential considering the potential future application of the biogel in infected DFU. Nisin concentrations were selected to allow testing in the following assays a nisin subinhibitory concentration (6.25 µg/mL), the average Minimum Inhibitory Concentration (MIC) for the four isolates under testing (25 µg/mL) and a 2× MIC value (50 µg/mL).

### 4.3. Storage Assay

Evaluation of storage influence on the antimicrobial activity of the nisin-biogel was performed using a spot-on-lawn assay, as previously described by Santos et al. [10]. Briefly, the four *S. aureus* strains used in this study were cultured in a non-selective brain-heart infusion (BHI) agar medium (VWR, Belgium) at 37 °C for 24 h. Afterward, bacterial suspensions at approximately 10^7^ CFU/mL were prepared in fresh BHI broth. Confluent bacterial lawns were produced by evenly spreading the 10^7^ CFU/mL bacterial suspensions onto BHI agar plates using sterile cotton swabs. Then, plates were dried for 10 min before applying a 3 µL dot of each nisin suspension to be tested. Plates were incubated at 37 °C for 24 h to allow bacterial growth before the measurement of inhibition halos. Assays were performed in triplicate and repeated every three months, for 24 months.

### 4.4. Cytotoxicity Assay

For evaluating the cytotoxic potential of the nisin-biogel, cryopreserved normal adult Human primary adherent Epidermal Keratinocytes (HEKa) (PCS-200-011, ATCC, Manassas, VA, USA) were cultured according to the producer’s instructions. Briefly, cells were cultured in Dermal Cell Basal Medium (PCS-200-030, ATCC, USA) supplemented with the Keratinocyte Growth Kit (PCS-200-040, ATCC, USA) in 75 cm^2^ cell culture flasks (Nunc; Thermo Fisher Scientific, Roskilde, Denmark), incubated at 37 °C in a humidified atmosphere of 5% CO_2_. Upon reaching a confluence of approximately 80%, cells were harvested using trypsin-EDTA (0.25%, Gibco; Thermo Fisher Scientific, Denmark), and viable cells were quantified after a 1:10 dilution in trypan blue (0.4%, Sigma-Aldrich, USA) using a Neubauer hemocytometer.

For the cytotoxicity assay, HEKa cells were seeded at a density of 10,000 cells per well in flat bottom polystyrene 96-well microplates (Nunc; Thermo Fisher Scientific, Denmark) and incubated at 37 °C in a humidified atmosphere of 5% CO_2_ for 48 h. Afterward, the growth medium was removed, and HEKa cells were exposed to 12 different suspensions of nisin, that varied in terms of concentration, delivery system, and storage duration, as described in Table 2. Testing wells were filled with 180 µL of growth medium plus 20 µL of the nisin suspensions under evaluation. As a positive control, cells were treated with doxorubicin hydrochloride (4 µM; Medac, Wedel, Germany). Solvent (0.02 M HCl) and delivery system (0.75% guar gum biogel) controls were also included in the assay.

After a 24 hours incubation at 37 °C in a humidified atmosphere of 5% CO_2_, *in vitro* cell viability was determined using the MTT Cell Proliferation Assay Kit (ab211091, Abcam, Cambridge, UK), according to manufacturer’s instructions. Briefly, growth medium was removed from all wells, and 50 µL of growth medium and 50 µL of 3-(4,5-dimethylthiazol-2-yl)-2,5-diphenyltetrazolium bromide (MTT) reagent were added into each well. Cells were then incubated at 37 °C for 3 h, after which 150 µL of MTT solvent was added into each well. Microplates were wrapped in foil and agitated on an orbital shaker for 15 min at room temperature. Cell viability was evaluated using a microplate reader (BGM LABTECH, Ortenberg, Germany) to measure optical density at a wavelength of 584 nm. Growth medium without cells was set as the blank control. Cell viability was expressed as a percentage relative to the untreated control (growth medium plus HEKa cells), which was set as being 100% viable. Assays were performed in triplicate.

### 4.5. Statistical Analysis

Statistical analysis was performed using the GraphPad Prism 5 Software for Windows. For storage assays, differences between delivery systems were evaluated using the *t*-test. Differences between storage temperatures were determined by analysis of variance using the one-way ANOVA followed by Tukey’s post-test. Finally, the influence of storage duration on nisin’s activity was analyzed using linear regression.

For cytotoxicity assays, the cell viability values presented by the suspensions under study were evaluated by analysis of variance using the one-way ANOVA followed by Dunnett’s post-test. A two-tailed *p* ≤ 0.05 was considered to be statistically significant in all the applied tests.

## 5. Conclusions

The nisin biogel demonstrated antimicrobial activity against *S. aureus* DFI isolates that are maintained after long-term storage at different temperatures. Furthermore, this nisin-biogel exhibited low cytotoxic activity towards human epidermal keratinocytes, either in fresh preparations or after long term storage. These results highlight the safety and stability of the antimicrobial nisin-biogel, supporting its potential use as a new topical complementary or alternative therapeutic approach in managing chronic DFIs.

## Figures and Tables

**Table 1:** Characteristics of the *S. aureus* DFI isolates used in this study.

Strain Characteristics	*S. aureus* DFI Isolates			
	Z5.2	Z16.1	Z21.3	Z25.2
*mecA*	−	+	+	−
*coa*	+	+	+	+
*spa*	+	+	+	+
*clfa*	+	+	+	+
*icaA*	+	+	+	+
*icaD*	+	+	+	+
*bap*	−	−	−	−
*pvl*	−	−	−	−
*agrI*	+	−	−	+
*agrII*	−	+	+	−
*tst*	−	−	−	−
ST	72	105	2599	944
CC	5	5	5	182
Antimicrobial resistance profile	−	Fox, Cip, Ery	Fox, Cyp, Ery	−
Biofilm-forming ability	Moderate at 24 h	Moderate at 24 h	Moderate at 24 h	Moderate at 48 h

Z—swab sample, *mecA*—methicillin resistance gene, *coa*—coagulase gene, *spa*—protein A gene, *clfa*—clumping factor a gene, *icaA* and *icaD*—adhesin genes, *bap*—biofilm associated protein gene, *pvl*—panton-valentine leucocidin gene, *agrI* and *agrII*—accessory regulators genes, *tst*—toxic shock syndrome toxin 1 gene, ST—sequence type, CC—clonal complex, Fox—cefoxitin, Cyp—ciprofloxacin, Ery—erythromycin, +—positive, −—negative.

**Table 2:** Concentrations, delivery system, and storage duration conditions of nisin to which HEKa cells were exposed in the cytotoxicity assays.

Nisin Concentration (µg/mL)	Delivery System	Storage Conditions
6.25	Sterile water	Freshly prepared
		Stored at 4 °C for 24 months
	Guar gum biogel	Freshly prepared
		Stored at 4 °C for 24 months
25	Sterile water	Freshly prepared
		Stored at 4 °C for 24 months
	Guar gum biogel	Freshly prepared
		Stored at 4 °C for 24 months
50	Sterile water	Freshly prepared
		Stored at 4 °C for 24 months
	Guar gum biogel	Freshly prepared
		Stored at 4 °C for 24 months
